# Family income is not significantly associated with T1w/T2w ratio in the Human Connectome Project in Development

**DOI:** 10.1162/imag_a_00021

**Published:** 2023-10-06

**Authors:** David G. Weissman, Graham L. Baum, Ashley Sanders, Maya L. Rosen, Deanna M. Barch, Katie A. McLaughlin, Leah H. Somerville

**Affiliations:** Department of Psychology, Harvard University, Cambridge, MA, United States; Department of Psychiatry, Washington University School of Medicine in St. Louis, St. Louis, MO, United States; Program in Neuroscience, Smith College, Northampton, MA, United States; Department of Psychological & Brain Sciences, Washington University in St. Louis, St. Louis, MO, United States; Department of Radiology, Washington University School of Medicine in St. Louis, St. Louis, MO, United States

**Keywords:** poverty, myelin, brain structure, cerebral cortex, socioeconomic status

## Abstract

Growing evidence indicates that brain development varies as a function of family socioeconomic status (SES). Numerous studies have demonstrated that children from low-SES backgrounds have thinner cortex than children from higher-SES backgrounds. A recent study in a large developmental sample found widespread associations between lower SES and greater cortical T1w/T2w ratio—thought to be an indirect proxy for cortical myelin. We evaluated the association of family income with cortical T1w/T2w ratio as a function of age in the Human Connectome Project in Development sample of 989 youth aged 8-21 years. We observed no associations between family income and T1w/T2w ratio that were significant after corrections for multiple comparisons at the region, network, or whole-brain level. Region of practical equivalence (ROPE) analyses were also consistent with the absence of an association between family income and T1w/T2w ratio. We discuss potential methodological sources of inconsistency between this and the previous study examining the same question. While the question of whether family income may influence cortical myelin development remains, these null results may indicate that the association between SES and cortical myelin development may not be as strong as with other aspects of brain structure.

## 1 Introduction

Growing evidence indicates that brain development varies as a function of family socioeconomic status (SES) (Hair et al., 2015; Hanson et al., 2013; Johnson et al., 2016; Mackey et al., 2015; Noble et al., 2015; Rakesh & Whittle, 2021). SES has been associated consistently with reduced thickness and surface area of cortical regions (Machlin et al., 2020; Noble et al., 2015; Sanders et al., 2022) and smaller volume of subcortical regions (Decker et al., 2020; Dufford et al., 2019; Ellwood-Lowe et al., 2018; Hair et al., 2015; Jenkins et al., 2020; Luby et al., 2013). Some studies have observed differences in the structural integrity of white matter tracts as a function of childhood SES (Ozernov-Palchik et al., 2019; Rosen, Sheridan, Sambrook, Meltzoff, et al., 2018), but investigation of differences in cortical myelination have been lacking. Recently, however, several groups have observed associations between SES and indices of cortical myelin content (Norbom et al., 2022; Ziegler et al., 2020), although the findings are in opposing directions. Characterizing the associations between SES and cortical myelin content and evaluating the extent to which any associations reflect deviations from typical age-related patterns may illuminate the precise nature of neurodevelopmental heterogeneity associated with socioeconomic disparities.

The ratio of T1-weighted to T2-weighted MRI images (T1w/T2w) can be used to indirectly estimate cortical myelin content (Glasser & Essen, 2011). T1w/T2w is correlated with both histological measures of myelin and other MRI indices of cortical myelin content (Glasser & Essen, 2011; Glasser et al., 2014; Shams et al., 2019). However, because MR signals are sensitive to properties like iron, cell density, and water content, in addition to myelin, the T1w/T2w ratio, while correlated with myelin content, represents a mix of these properties (Baum et al., 2022; Carey et al., 2018; Glasser et al., 2022). T1w/T2w ratio increases from childhood to adulthood, following the opposite trajectory from cortical thickness (Baum et al., 2022). Decreases in cortical thickness from childhood through early adulthood are a normative developmental process (Frangou et al., 2022). However, recent work has suggested that the developmental trajectory of cortical thinning showing reductions over time actually reflects greater myelination of the cortex, rather than thinning of the gray matter due to the influence of myelination on the contrast between gray and white matter in the cortex (Natu et al., 2019), a pattern long postulated to contribute to age-related cortical thinning (Sowell et al., 2004). Age-related patterns of T1w/T2w ratio across the brain appear similar with and without controls for cortical thickness, suggesting that T1w/T2w myelin and cortical thickness reflect dissociable mechanisms of structural neurodevelopment (Baum et al., 2022). Thus, the association between SES and T1w/T2w ratio may be similarly dissociable from the association between SES and cortical thickness and surface area.

Differences in cortical myelin content may be an age-invariant consequence of low SES as has been suggested for other measures of structural neurodevelopment (Rakesh et al., 2023), or it may reflect altered neurodevelopment, and thus impact the trajectory of myelin development. The two existing studies on this topic have produced conflicting findings. One study using an accelerated longitudinal design and magnetization transfer—a different method to quantify cortical myelin content—found that higher neighborhood-level economic disadvantage was associated with slower myelin growth (Ziegler et al., 2020). Another recent study in a large (n = 502) developmental sample aged 3-21 years old found widespread associations between lower SES (measured as a composite of family income, parental education, and parental occupation) and greater T1w/T2w ratio across the brain, independent of age, suggesting that low SES was associated either with greater cortical myelin content across development but not with differences in the rate of myelination (Norbom et al., 2022). A third study, once again using magnetization transfer, found overall higher myelin content in the sensorimotor network but lower myelin content in the temporal lobe associated with childhood SES in older adults (Loued-Khenissi et al., 2022). These studies probed different aspects of the SES construct and inferred cortical myelination based on different neuroimaging metrics. Thus, while discrepancies in the findings are not surprising, they nonetheless suggest that there may not be a broad association between SES and cortical myelin development that is robust to these conceptual and methodological differences, and that further investigation is necessary to clarify which aspects of SES influence which measures of cortical myelination development.

While cross-sectional data are limited with respect to the conclusions that can be drawn about developmental processes, statistical methods that characterize age-related patterns based on multivariate patterns (e.g., “Brain age”) can be useful in making neurodevelopmental inferences with considerable predictive accuracy (Cole et al., 2017; Dosenbach et al., 2010; Franke et al., 2010). In this study, we use gaussian process regression to provide statistical inferences about whether T1w/T2w ratio development is accelerated or delayed with respect to the age-typical localized T1w/T2w ratio, based on a model developed in a training dataset.

We examined the association between family income, one measure of SES, and T1w/T2w myelin content in a sample of 989 youth aged 8-21 years. We extend the prior studies on this topic that have produced conflicting findings by examining whether family income is associated with deviations from normative, nonlinear age curves in T1w/T2w ratio. Although conducted in a cross-sectional sample, this analytic approach evaluates whether associations of family income with T1w/T2w ratio reflect accelerated or delayed developmental trajectories.

## 2 Methods

All methods and analyses were preregistered (https://osf.io/duvbj).

### 2.1 Sample

The present sample consists of 925 8-21 year old participants (50.3% female) in the Human Connectome Project in Development (HCP-D). Participants were recruited across four sites: Harvard University, University of California-Los Angeles, University of Minnesota, and Washington University in St. Louis. Exclusion criteria for recruitment included (i) premature birth (<37 weeks gestation); (ii) serious neurological condition (e.g., stroke, cerebral palsy); (iii) serious endocrine condition (e.g., precocious puberty, untreated growth hormone deficiency); (iv) long-term use of immunosuppressants or steroids; (v) any history of serious head injury; (vi) hospitalization >2 days for certain physical or psychiatric conditions or substance use; (vii) treatment >12 months for psychiatric conditions; (viii) claustrophobia; or (ix) pregnancy. Participants provided written informed consent and assent and parents of participants under 18 years provided written informed consent for their child’s participation. All procedures were approved by a central Institutional Review Board administered at Washington University in St. Louis (IRB #201603135) and were performed in accordance with the ethical standards as outlined in the 1964 Declaration of Helsinki.

Participants were included if their T1w/T2w ratio maps were of sufficient quality based on manual inspection of scalar properties and the accuracy of image segmentation, as determined by trained experts in the HCP-D consortium (Elam et al., 2021). Following cortical surface reconstruction, a single experienced individual performed a “SurfaceQC” review of the white and gray matter surface placement, informed by the T1w/T2w ratio maps (Elam et al., 2021; Glasser & Essen, 2011). Participants with more than minor (focal) issues were flagged for possible future editing and excluded from the cohort analyzed for the current study. This “SurfaceQC” review of the HCP-D data revealed some degradation of the accuracy of surface placement relative to expectations established by the HCP Young Adult project, which were traced to artifacts in the longer echos. Therefore, to reduce the prevalence of surface segmentation errors in this developmental sample, we used the mean of the shortest two echos (i.e., excluded the longest two of four echos) as the T1w input to the HCP Pipelines (Elam et al., 2021).

### 2.2 Measures

#### 2.2.1 Family income

Family income was operationalized as the natural log of the income-to-needs ratio, which is calculated by dividing parent-reported family income by the 2017 federal poverty line based on the family size reported by the parent. The estimate of family income was entered into a text box in response to the prompt, “Please state your TOTAL COMBINED FAMILY INCOME for the past 12 months. This should include income (before taxes and deductions) from all sources, wages, rent from properties, social security, disability and/or veteran’s benefits, unemployment benefits, workman’s compensation, help from relatives (including child payments and alimony), and so on.” To limit the influence of incomes at the extreme ends of the distribution, incomes greater than $300,000 were recoded as $300,000 (n = 71). Incomes less than $15,000 were recoded as $15,000 (n = 44). Consistent with prior work on childhood SES and neurodevelopment (Noble et al., 2015; Rosen, Sheridan, Sambrook, Peverill, et al., 2018), we used the natural log of income-to-needs ratio to reflect that associations of income with neural outcomes are non-linear with stronger associations at the lower end of the income distribution.

For supplemental analyses that were not part of the original preregistration (https://osf.io/duvbj), we also conducted analyses using maternal education as a measure of SES. Maternal education was defined as the highest educational level achieved by the child’s mother. We also computed a composite measure of SES by standardizing both parental education and log income-to-needs ratio and computing the average.

#### 2.2.2 T1w/T2w ratio

T1w/T2w ratio was estimated by taking the ratio between high-resolution (0.8 mm isotropic) T1w and T2w voxels mapped to the cortical surface using methods developed by the HCP consortium (Glasser & Essen, 2011; Glasser et al., 2013, 2014; Marcus et al., 2011). Division of the T1w image by the T2w image mathematically cancels the signal intensity bias related to the sensitivity profile of the radio frequency receiver coils, and enhances the contrast of cortical myelin content (Glasser & Essen, 2011). We also applied an empirically validated “pseudo-transmit field” correction to mitigate B1+ bias in individual T1w/T2w ratio maps, thereby reducing potentially spurious age-related differences in T1w/T2w ratio (Baum et al., 2022; Glasser et al., 2022).

As described in detail in previous publications (Baum et al., 2022; Glasser et al., 2022), the B1+ correction relies on computing a pseudo-transmit field. First, a reference T1w/T2w map was generated at the group level by finding the scaling between the group average pseudo-transmit field and group average T1w/T2w map that minimizes the correlated left-right differences between the two maps (i.e., the clearly spurious left-right asymmetries). This reference group map was used to correct the individual maps. For the individual correction, the pseudo-transmit map was scaled to minimize the correlated differences between the individual’s T1w/T2w map and the reference T1w/T2w map and the pseudo-transmit map (which includes all differences, not simply left-right ones, and is more robust at the individual level). Before estimating this correction, any residual B1– effects because of subject head motion between the T1w and T2w images were also removed using the scanner-computed B1– receive field. The pseudo-transmit field requires regularization by thresholding regions of T2*-related signal loss combined with spatial smoothing (with compensation for intensity changes induced by smoothing); it is then scaled to equal 1 at the value where the GRE/SE ratio corresponds to the flip angle prescribed by the scanner, a reference value that is determined at the group level.

Individual T1w/T2w ratio maps were parcellated into regions based on the HCP multimodal atlas (Glasser et al., 2016) and into networks based on the Cole-Anticevic atlas (Ji et al., 2019). The PostFreeSurfer pipeline produced cortical surface models in GIFTI format and surface-related data in CIFTI format, and each subject’s cortical surface was then registered to a common 32k_FS_LR mesh using “MSMAll” areal-feature-based cortical surface registration, which is a multimodal registration constrained by cortical T1w/T2w maps and resting-state network maps (Glasser et al., 2016).

#### 2.2.3 Modeling deviations from normative T1w/T2w development

We applied normative modeling using gaussian process regression to provide statistical inferences at the level of the individuals with respect to normative patterns of T1w/T2w ratio development. A key advantage of this approach is that in addition to fitting potentially non-linear relationships between age and T1w/T2w ratio, it also provides regional estimates of the expected variation in the relationship between age and T1w/T2w ratio (normative variance) as well as estimates of uncertainty in this variance. Both normative variance and uncertainty are learned from a training subset. Then, for each participant (*i*) in the test subset, we generate the predicted brain feature (*ŷ_ij_*) and combine it with the true value of the brain feature (*y_ij_*), the predictive uncertainty (*σ_ij_*), and the normative variance (*σ_nj_*) to create a z-score that quantifies deviation from normative neurodevelopment (Marquand et al., 2019). Unlike a residual, which is the difference between the predicted and actual value (*ŷ_ij_ - y_ij_*), the difference score is computed as:

$$\frac{\text{ŷij}-\text{yij}}{\sqrt{\sigma \text{ij}+\sigma \text{nj}}}$$

We then tested whether deviations from normative T1w/T2w ratio development are associated with log income-to-needs ratio.

### 2.3 Analyses

For all analyses, generalized additive models with age splines were used (Wood, 2011) using the mgcv package in R (Wood, 2017) to estimate both linear and nonlinear associations between log income-to-needs ratio and T1w/T2w ratio development, both continuous variables. In the first analysis, log income-to-needs ratio was the independent variable and T1w/T2w ratio was the dependent variable. Participant age, sex, scanner, and seven nuisance regressors for B1+ correction (the scanner transmit voltage, the mean of the pseudotransmit map, T2* dropout threshold, smoothing FWHM, correction factor for smoothing’s effect on the pseudotransmit field’s intensities, the slope parameter of the correction, and a corrected T1w/T2w lateral ventricular CSF regressor) were included as covariates. The correlations between those seven nuisance regressors and log income-to-needs ratio ranged from r = -.16 to r = .03. In the second analysis, the dependent variable was deviations from normative T1w/T2w development, a continuous variable in arbitrary units. Covariates were participant sex and scanner type.

Analyses were conducted in parallel for each region in the brain, parcellated according to the HCP-multimodal atlas and each network in the brain, parcellated according to the Cole-Anticevic atlas. Holm’s adjustment (Holm, 1979) was used for multiple comparison correction across regions and networks. Bayesian parameter estimation using the brms package in R (Bürkner et al., 2017) was used to guide inference on the likelihood that observed null age effects reflected a true underlying null distribution using a region of practical equivalence (ROPE) approach (Kruschke, 2011). For the ROPE analyses, a standardized regression coefficient smaller than |.06| was considered practically equivalent to 0. This effect size was chosen because smaller effects are unlikely to be particularly meaningful at the population level or replicable, even in large samples (Marek et al., 2022). A sample size of around 9,500 is required to detect an effect of that size with multiple comparison corrections or a sample size of 2,200 for uncorrected *p* < .05. As noted above, all analyses were repeated using maternal education as a second metric of SES, and for a third time using a composite measure of SES. These analyses were not pre-registered but followed the identical structure of pre-registered analyses for income-to-needs. All analytic codes are available at https://github.com/dgweissman/hcpd_adversity.

## 3 Results

The sample had a wide (8-22 years) and uniform age distribution (*Mean* = 14.40, *SD* = 3.99). While the income distribution of the sample was higher (median of $110,000 per year) than what would be nationally representative, the distribution of income-to-needs ratio was quite wide (0.12-14.7). Fifty-seven participants (6.1%) had incomes below the federal poverty line, and 148 participants (16%) had incomes below 200% of the federal poverty line. A range of education levels were also represented in the sample (see Table 1).

**Table 1.** Participant demographics.

	n		%	
Sex				
Female	466		50.4	
Male	459		49.6	
Race				
Asian	99		10.7	
Black/African American	141		15.2	
Native American/Alaska Native	12		1.3	
Native Hawaiian/Pacific Islander	4		0.4	
White	583		63.0	
More than one race	67		7.2	
Unknown or not reported	19		2.1	
Highest parental education level				
Less than high school	76		8.2	
High school	174		18.8	
Some college	283		30.6	
Bachelor’s degree	215		23.2	
Postgraduate degree	125		13.5	

	Mean	SD	Min	Max
Age	14.4	3.99	8.01	22.0
Income-to-needs ratio	4.95	3.08	0.12	14.7

**Fig. 1.**
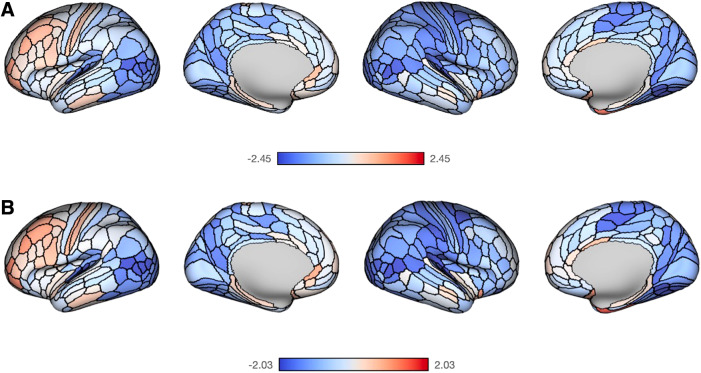
Associations between family income and T1w/T2w ratio. (A) Maps represent t-statistics across cortical parcellations of the association between log income-to-needs ratio and T1w/T2w ratio based on the model: Regional T1w/T2w ~ log Income-to-needs-ratio + s(Age) + Sex + Site + “B1+” bias correction covariates, where s(Age) is a generalized additive age spline. (B) Maps represent t-statistics across cortical parcellations of the association between log income-to-needs ratio and the regional myelin deviation scores based on normative modeling, controlling for participants’ actual age.

The associations between family income and T1w/T2w ratio were mostly weakly negative but were not statistically significant. There were no associations between SES, measured by log income-to-needs ratio, and T1w/T2w ratio that were significant after corrections for multiple comparisons at the region (Glasser parcels, Fig. 1), network (Table 2), or whole-brain level (*B* = -.00263, *SE* = .00259, *t* = -1.02, *p* = .310). The strongest negative associations between SES and T1w/T2w ratio were observed in the right ventromedial visual cortex (*t* = -2.9, *uncorrected p* = .004) and left medial belt (*t* = -2.8, *uncorrected p* = .005). Notably, if the B1+ covariates were not included in analyses, the association between log income-to-needs ratio and whole-brain T1w/T2w ratio was larger though still only marginally significant (*B* = -.00583, *SE* = .00311, *t* = -1.88, *p* = .061). Neither parcel- nor network-level associations between log income-to-needs ratio and T1w/T2w ratio were significant after multiple comparison corrections, even without inclusion of B1+ covariates.

**Table 2.** Association between family income and T1w/T2w ratio by network.

Cortical network	t-statistic	*p*-value
Visual1	-1.14	.26
Visual2	-1.74	.08
Somatomotor	-1.26	.21
Cingulo Opercular	-0.81	.42
Dorsal Attention	-1.15	.25
Language	-0.76	.45
Frontoparietal	-0.55	.58
Auditory	-1.82	.07
Default	-0.59	.56
Posterior Multimodal	-1.93	.053
Ventral Multimodal	0.25	.8
Orbito Affective	0.74	.46

Note: Family income is operationalized as the natural log of the income-to-needs ratio.

Based on ROPE analyses, the majority (>76%) of posterior estimates of the association between log income-to-needs ratio and network-level T1w/T2w ratio fell within ROPE intervals considered effectively zero for all networks. The majority (>50%) of posterior estimates of the association between log income-to-needs ratio and parcel-level T1w/T2w ratio fell within ROPE intervals considered effectively zero for 355 out of 360 parcels. For 5 parcels (including ventromedial visual cortex and left medial belt), the results were inconclusive. While not significantly different from 0, their association with log income-to-needs ratio also cannot be considered practically equivalent to 0.

Similarly, associations with SES measured by maternal education were mostly weak, negative, and none were statistically significant after corrections for multiple comparisons at the region, network, or whole-brain level (*B = *-.00131, *SE* = .00089, *t* = -1.47, *p* = .141). The strongest negative associations between SES and T1w/T2w ratio were observed in the right dorsal anterior cingulate cortex (Area 24dd; *t* = -3.05, *uncorrected p* = .002) and left ventral visual cortex (VVC; *t* = -2.66, *uncorrected p* = .008). Notably, if the B1+ covariates were not included in analyses, the association between maternal education and whole-brain T1w/T2w ratio was considerably larger and significant (*B= *-.00224, *SE* = .00107, *t* = -2.10, *p* = .036). However, neither parcel- nor network-level associations between maternal education and T1w/T2w ratio were significant after multiple comparison corrections, even without inclusion of B1+ covariates. Based on ROPE analyses, the majority (>70%) of posterior estimates of the association between maternal education and network-level T1w/T2w ratio fell within ROPE intervals considered effectively 0 for all networks. For 18 parcels (including right dorsal anterior cingulate cortex and left ventral visual cortex), the results were inconclusive. While not significantly different from 0, their association with log income-to-needs ratio also cannot be considered practically equivalent to 0.

There were no significant associations between family income or maternal education and deviations from normative T1w/T2w ratio development. The patterns of mostly weakly negative associations—in the direction of accelerated T1w/T2w ratio development among lower income participants—were very similar to the main effects of family income (Fig. 1).

As when conducted separately, associations with SES measured by a composite measure of SES created by standardizing and then averaging parental education and log income-to-needs ratio were mostly weak, negative, and none were statistically significant after corrections for multiple comparisons at the region, network, or whole-brain level (see Supplemental Materials).

## 4 Discussion

Overall, despite having a large sample of almost one thousand children, adolescents, and young adults with a wide distribution of age and family income, strong data acquisition and analysis pipelines, and analyses that included bias field corrections, we did not observe significant associations between family income and T1w/T2w ratio. Thus, our inferences are inconsistent with those based on an earlier large multisite neuroimaging study. However, the overall pattern of uncorrected associations between family income and T1w/T2w ratio in the HCP-D sample demonstrated a similar spatial pattern across the brain to what was observed in relation to an SES composite in a previous study by Norbom and colleagues (2022). Because both studies rely on large, public data sets with their own unique standardized processing pipelines, and as the current study was preregistered before publication of Norbom et al. (2022), some methodological differences may at least partially account for this discrepancy.

First, Norbom and colleagues used a composite measure of SES, consisting of family income (log total family income), parental education, and parental occupation. When they examined these measures separately, they similarly found no significant association between family income and T1w/T2w ratio. Conversely, they found widespread associations between lower parental education and greater T1w/T2w ratio across the entire brain (Norbom et al., 2022, Supplementary Figure 2). Finally, they found associations between parental occupation and T1w/T2w ratio that were concentrated in visual and association cortices, thereby contributing to the regional specificity seen in the main analyses using the composite measure of SES. Thus, the main discrepancy between the findings in these analyses and those observed by Norbom and colleagues was the absence of widespread significant associations between parental education and T1w/T2w ratio content in the current study.

Another important methodological difference between the present study and the study by Norbom and colleagues is the use of correction for B1+ artifact. As noted in recent work by Glasser and colleagues (2022), T1w/T2w ratio maps contain residual radiofrequency transmit field (B1+) biases, which may be correlated with variables like body-mass-index (BMI), that are, in turn, correlated with SES. It is therefore possible that by (appropriately) correcting for B1+ artifact, we diminished the strength of the associations between family income and T1w/T2w ratio that might reflect other factors that are related to family income but not cortical myelin content. Indeed, in a supplementary analysis examining the association between log income-to-needs ratio and whole-brain T1w/T2w ratio, excluding the correction for B1+ artifact, the observed effect was over twice as large but still only marginally significant.

Finally, Norbom and colleagues used vertex-wise data instead of a cortical parcellation as was applied in this study and controlled for genetic ancestry. Our use of a parcellation reduced the number of analyses and therefore the penalty for multiple comparisons, which should only increase the likelihood of detecting a significant association given the pattern of widespread weak associations. Controlling for genetic ancestry, as was done by Norbom and colleagues, addresses the issue of whether inherited characteristics of ancestry contribute to differences in brain structure. However, no data on genetic ancestry are currently available in the HCP-D sample in order to include such a variable, and it is our view that using individual-level racial categories as variables of interest or covariates presumes a biological basis for these racial categories that is not supported by evidence (see Helms et al., 2005 for extensive discussion of this issue). We consider these methodological discrepancies, while notable, less likely to have contributed to the discrepancy in the strength of the observed associations than the measures of SES used and B1+ artifact correction. Sensitivity analyses revealed that inclusion of the B1+ artifact covariates in analyses substantially reduced the effect size estimates of the associations between SES indicators and the T1w/T2w ratio.

We also failed to find significant associations between low family income and slower T1w/T2w ratio growth observed in a previous longitudinal study (Ziegler et al., 2020). In fact, the nonsignificant findings observed in this study were in the direction of accelerated development, opposite the direction of those observed in the earlier study. Several methodological differences may have accounted for these discrepancies, including the use of longitudinal methods vs. normative models to estimate accelerated or delayed neurodevelopment, the use of neighborhood disadvantage vs. individual family income and parental education as measures of SES, and the use of magnetization transfer vs. T1w/T2w ratio to quantify cortical myelin content. It therefore appears clear that there is not a broad association between SES and cortical myelin development that is robust to these conceptual and methodological differences. Further investigation would therefore be necessary to clarify what aspects of SES, experienced at what ages, may or may not shape the trajectory of cortical myelin development, and to evaluate whether findings replicate across methodologies for quantifying cortical myelination with appropriate controls for potential artifact and methodological confounds.

This sample, while large, may not be large enough to detect significant brain-wide associations between SES and T1w/T2w ratio. Brain-wide associations with individual difference characteristics tend to be quite small, and therefore sample sizes in the thousands are required to reliably detect them (Marek et al., 2022). Nonetheless, in the same Human Connectome Project in Development sample reported on here, low maternal education and low income were associated with significantly lower cortical thickness across multiple brain networks (Sanders et al., 2022), consistent with earlier findings (Noble et al., 2015). Therefore, there is some suggestion that the association between SES and cortical thickness is dissociable from and stronger than the association between SES and cortical myelin as measured by the T1w to T2w ratio.

In conclusion, we did not find evidence that family income is significantly related to T1w/T2w ratio, suggesting that, in early life, there may not be a broad association between SES and cortical myelin development that is robust and consistent across measures of SES and methodological decisions, even in large samples.

## Supplementary Material

Supplementary Material

## Data Availability

All analytic codes are available at https://github.com/dgweissman/hcpd_adversity. All data from the Human Connectome Project in Development used in this study are publicly available from the NIMH Data Archive (https://nda.nih.gov/).
