# Supplementary material for: Family income is not significantly associated with T1w/T2w ratio in the Human Connectome Project in Development
Source: Imaging Neurosci (Camb). 2023 Oct 6;1:imag-1-00021. doi: 10.1162/imag_a_00021 (PMC11242614; doi:10.1162/imag_a_00021)
Supplement: Supplementary Material [file imag_a_00021-supp.pdf]

Supplemental Information

Composite SES was computed by standardizing log income-to-needs ratio and parental education and computing the mean of those two measures. The association between composite SES and T1w/T2w ratio was not significant (*B*= -.00310, *SE*=.00248, *t*=-1.25, *p*=.212).

Table S1: Associations of parental education and composite SES with T1w/T2w Ratio by Network

	Parental Education		SES Composite	
Cortical Network	*t*	*p*	*t*	*p*
Visual1	-0.51	.61	-0.91	.36
Visual2	-1.47	.14	-1.73	.08
Somatomotor	-1.61	.11	-1.42	.16
Cingulo Opercular	-1.37	.17	-1.05	.29
Dorsal Attention	-1.23	.22	-1.20	.23
Language	-1.63	.10	-1.17	.24
Frontoparietal	-1.17	.24	-0.82	.41
Auditory	-1.71	.09	-1.82	.07
Default	-1.37	.17	-0.95	.34
Posterior Multimodal	-1.32	.19	-1.78	.08
Ventral Multimodal	-0.91	.37	-0.23	.82
Orbito Affective	0.68	.49	0.75	.45

Table S2. All t-statistics and p-values of associations between SES and T1w/T2w ratio at each parcel

SES Measure	Log Income-to-needs ratio			Parental education			SES Composite		
**Glasser Parcel**	*t*	*p-uncor*	*p-cor*	*t*	*p-uncor*	*p-cor*	*t*	*p-uncor*	*p-cor*
Right V1	-0.99	0.322	1	-0.21	0.832	1.00	-0.73	0.465	1.00
Right MST	-2.38	0.018	1	-0.96	0.338	1.00	-1.86	0.063	1.00
Right V6	-1.41	0.159	1	-0.75	0.451	1.00	-1.17	0.244	1.00
Right V2	-1.45	0.147	1	-1.00	0.319	1.00	-1.38	0.168	1.00
Right V3	-1.62	0.106	1	-1.37	0.170	1.00	-1.63	0.103	1.00
Right V4	-2.20	0.028	1	-1.79	0.074	1.00	-2.25	0.025	1.00
Right V8	-2.27	0.023	1	-2.55	0.011	1.00	-2.81	0.005	1.00
Right 4	-1.90	0.058	1	-1.71	0.087	1.00	-1.76	0.079	1.00
Right 3b	-1.18	0.240	1	-0.77	0.442	1.00	-0.87	0.383	1.00
Right FEF	-2.28	0.023	1	-1.94	0.053	1.00	-2.08	0.038	1.00
Right PEF	-0.43	0.666	1	-0.36	0.722	1.00	-0.30	0.766	1.00
Right 55b	-1.49	0.138	1	-1.10	0.273	1.00	-1.22	0.224	1.00
Right V3A	-0.42	0.675	1	-0.30	0.762	1.00	-0.40	0.687	1.00
Right RSC	-0.64	0.526	1	-1.21	0.226	1.00	-0.88	0.377	1.00

Right POS2	-1.47	0.142	1	-0.79	0.427	1.00	-1.25	0.211	1.00
Right V7	-0.60	0.550	1	-0.24	0.813	1.00	-0.41	0.678	1.00
Right IPS1	-0.28	0.779	1	-0.65	0.517	1.00	-0.60	0.547	1.00
Right FFC	-1.65	0.098	1	-1.21	0.228	1.00	-1.48	0.140	1.00
Right V3B	-1.17	0.244	1	-0.60	0.549	1.00	-1.01	0.311	1.00
Right LO1	-1.75	0.081	1	-0.51	0.607	1.00	-1.28	0.201	1.00
Right LO2	-1.53	0.127	1	-1.62	0.106	1.00	-1.83	0.067	1.00
Right PIT	-1.64	0.102	1	-2.15	0.031	1.00	-2.18	0.029	1.00
Right MT	-1.68	0.093	1	-0.49	0.626	1.00	-1.25	0.213	1.00
Right A1	-2.09	0.037	1	-0.69	0.490	1.00	-1.32	0.186	1.00
Right PSL	-1.63	0.103	1	-0.28	0.782	1.00	-0.94	0.346	1.00
Right SFL	-0.40	0.693	1	-1.17	0.241	1.00	-0.65	0.517	1.00
Right PCV	-1.21	0.226	1	-0.55	0.581	1.00	-0.94	0.348	1.00
Right STV	-1.35	0.178	1	-1.05	0.294	1.00	-1.27	0.206	1.00
Right 7Pm	-1.01	0.314	1	-0.31	0.754	1.00	-0.78	0.438	1.00
Right 7m	-1.85	0.065	1	-1.10	0.271	1.00	-1.61	0.109	1.00
Right POS1	-1.60	0.111	1	-1.40	0.163	1.00	-1.57	0.118	1.00
Right 23d	-0.81	0.416	1	-1.30	0.193	1.00	-1.05	0.292	1.00
Right v23ab	-0.96	0.337	1	-1.20	0.229	1.00	-1.17	0.241	1.00
Right d23ab	-0.81	0.420	1	-1.18	0.238	1.00	-0.97	0.333	1.00
Right 31pv	-0.36	0.719	1	-0.51	0.611	1.00	-0.50	0.617	1.00
Right 5m	-1.94	0.053	1	-2.40	0.016	1.00	-2.24	0.025	1.00
Right 5mv	-1.36	0.175	1	-1.34	0.181	1.00	-1.38	0.167	1.00
Right 23c	-1.36	0.173	1	-1.69	0.091	1.00	-1.63	0.103	1.00
Right 5L	-1.93	0.054	1	-2.42	0.016	1.00	-2.41	0.016	1.00
Right 24dd	-2.13	0.034	1	-3.05	0.002	0.86	-2.67	0.008	1.00
Right 24dv	-1.95	0.051	1	-2.53	0.011	1.00	-2.33	0.020	1.00
Right 7AL	-1.71	0.088	1	-2.00	0.046	1.00	-2.03	0.042	1.00
Right SCEF	-0.44	0.661	1	-0.90	0.367	1.00	-0.56	0.577	1.00
Right 6ma	-0.82	0.415	1	-1.26	0.210	1.00	-0.87	0.382	1.00
Right 7Am	-1.05	0.293	1	-1.39	0.166	1.00	-1.29	0.197	1.00
Right 7PL	0.07	0.943	1	-0.02	0.986	1.00	0.13	0.897	1.00
Right 7PC	-1.37	0.171	1	-1.01	0.311	1.00	-1.28	0.201	1.00
Right LIPv	-1.55	0.122	1	-1.52	0.130	1.00	-1.75	0.081	1.00
Right VIP	-1.42	0.156	1	-1.80	0.072	1.00	-1.72	0.086	1.00
Right MIP	-1.56	0.120	1	-1.21	0.225	1.00	-1.59	0.111	1.00
Right 1	-1.41	0.160	1	-1.35	0.177	1.00	-1.31	0.189	1.00
Right 2	-1.97	0.050	1	-1.69	0.092	1.00	-1.90	0.058	1.00
Right 3a	-0.98	0.328	1	-0.98	0.326	1.00	-0.89	0.375	1.00
Right 6d	-2.04	0.042	1	-2.10	0.036	1.00	-2.20	0.028	1.00

Right 6mp	-1.88	0.061	1	-1.83	0.068	1.00	-1.81	0.070	1.00
Right 6v	-1.45	0.146	1	-1.24	0.214	1.00	-1.45	0.147	1.00
Right p24pr	-0.69	0.491	1	-1.72	0.085	1.00	-1.15	0.251	1.00
Right 33pr	0.61	0.539	1	-0.05	0.962	1.00	0.56	0.575	1.00
Right a24pr	-0.08	0.939	1	-1.03	0.301	1.00	-0.45	0.651	1.00
Right p32pr	-0.56	0.576	1	-1.74	0.082	1.00	-1.05	0.295	1.00
Right a24	0.04	0.970	1	-0.87	0.386	1.00	-0.34	0.737	1.00
Right d32	-0.59	0.555	1	-0.98	0.329	1.00	-0.74	0.463	1.00
Right 8BM	-0.71	0.481	1	-0.77	0.443	1.00	-0.65	0.514	1.00
Right p32	-0.21	0.830	1	-0.79	0.431	1.00	-0.40	0.686	1.00
Right 10r	-0.57	0.569	1	-0.92	0.356	1.00	-0.64	0.521	1.00
Right 47m	-2.02	0.044	1	-1.75	0.080	1.00	-1.89	0.060	1.00
Right 8Av	-1.17	0.240	1	-1.58	0.114	1.00	-1.33	0.185	1.00
Right 8Ad	-0.95	0.341	1	-1.02	0.307	1.00	-1.01	0.313	1.00
Right 9m	-0.35	0.726	1	-0.51	0.609	1.00	-0.38	0.707	1.00
Right 8BL	-0.18	0.858	1	-0.64	0.525	1.00	-0.31	0.756	1.00
Right 9p	0.21	0.830	1	-0.20	0.845	1.00	0.10	0.924	1.00
Right 10d	0.18	0.859	1	-0.31	0.759	1.00	0.08	0.938	1.00
Right 8C	-0.73	0.464	1	-1.30	0.195	1.00	-1.00	0.316	1.00
Right 44	-0.80	0.426	1	-0.98	0.326	1.00	-0.93	0.354	1.00
Right 45	-0.64	0.520	1	-1.01	0.313	1.00	-0.90	0.366	1.00
Right 47l	-0.91	0.362	1	-1.61	0.108	1.00	-1.31	0.192	1.00
Right a47r	-0.40	0.687	1	-1.07	0.284	1.00	-0.70	0.483	1.00
Right 6r	-1.45	0.148	1	-1.18	0.238	1.00	-1.37	0.170	1.00
Right IFJa	-1.10	0.271	1	-0.81	0.416	1.00	-0.93	0.351	1.00
Right IFJp	-1.23	0.220	1	-2.03	0.043	1.00	-1.93	0.054	1.00
Right IFSp	-1.25	0.211	1	-1.37	0.171	1.00	-1.37	0.170	1.00
Right IFSa	-1.27	0.205	1	-1.61	0.108	1.00	-1.57	0.118	1.00
Right p9-46v	-0.50	0.619	1	-1.17	0.241	1.00	-0.93	0.353	1.00
Right 46	-0.38	0.707	1	-0.81	0.419	1.00	-0.54	0.590	1.00
Right a9-46v	-0.30	0.764	1	-0.95	0.344	1.00	-0.61	0.543	1.00
Right 9-46d	-0.41	0.682	1	-0.28	0.778	1.00	-0.27	0.787	1.00
Right 9a	0.21	0.835	1	-0.01	0.992	1.00	0.23	0.820	1.00
Right 10v	-0.54	0.588	1	-1.94	0.052	1.00	-1.35	0.177	1.00
Right a10p	-0.36	0.717	1	-1.11	0.268	1.00	-0.66	0.510	1.00
Right 10pp	-0.31	0.760	1	-1.65	0.099	1.00	-0.93	0.352	1.00
Right 11l	-0.58	0.561	1	-1.66	0.098	1.00	-1.09	0.278	1.00
Right 13l	-0.43	0.665	1	-1.03	0.305	1.00	-0.66	0.510	1.00
Right OFC	-0.64	0.520	1	-1.52	0.128	1.00	-1.01	0.315	1.00
Right 47s	-1.06	0.289	1	-2.11	0.035	1.00	-1.66	0.098	1.00

Right LIPd	-1.10	0.271	1	-0.52	0.605	1.00	-0.95	0.343	1.00
Right 6a	-1.37	0.172	1	-1.61	0.108	1.00	-1.47	0.141	1.00
Right i6-8	-1.00	0.317	1	-1.63	0.103	1.00	-1.29	0.196	1.00
Right s6-8	-0.99	0.323	1	-1.30	0.195	1.00	-1.10	0.270	1.00
Right 43	-0.98	0.327	1	-0.93	0.351	1.00	-0.91	0.361	1.00
Right OP4	-1.73	0.083	1	-0.96	0.339	1.00	-1.48	0.140	1.00
Right OP1	-2.18	0.030	1	-1.49	0.137	1.00	-1.89	0.059	1.00
Right OP2-3	-1.15	0.249	1	-1.20	0.230	1.00	-1.17	0.244	1.00
Right 52	-2.29	0.022	1	-1.96	0.050	1.00	-2.29	0.022	1.00
Right RI	-2.18	0.030	1	-1.44	0.149	1.00	-1.91	0.057	1.00
Right PFcm	-1.89	0.059	1	-1.25	0.211	1.00	-1.69	0.092	1.00
Right PoI2	-0.23	0.816	1	-0.41	0.685	1.00	-0.28	0.783	1.00
Right TA2	-1.42	0.157	1	0.17	0.865	1.00	-0.59	0.556	1.00
Right FOP4	-0.99	0.325	1	-1.46	0.144	1.00	-1.25	0.212	1.00
Right MI	-1.16	0.245	1	-1.23	0.220	1.00	-1.32	0.186	1.00
Right Pir	0.64	0.521	1	1.24	0.215	1.00	0.92	0.358	1.00
Right AVI	-1.02	0.307	1	-0.98	0.325	1.00	-0.93	0.350	1.00
Right AAIC	0.82	0.412	1	1.22	0.223	1.00	1.36	0.173	1.00
Right FOP1	-0.44	0.661	1	-0.58	0.563	1.00	-0.56	0.577	1.00
Right FOP3	-0.63	0.526	1	-0.67	0.502	1.00	-0.73	0.463	1.00
Right FOP2	-0.83	0.406	1	-0.89	0.374	1.00	-0.87	0.387	1.00
Right PFt	-2.12	0.034	1	-1.76	0.080	1.00	-1.98	0.048	1.00
Right AIP	-1.45	0.148	1	-0.91	0.365	1.00	-1.19	0.236	1.00
Right EC	-0.59	0.553	1	-2.12	0.035	1.00	-1.43	0.154	1.00
Right PreS	0.05	0.962	1	-0.01	0.992	1.00	-0.07	0.945	1.00
Right H	0.14	0.892	1	-1.25	0.212	1.00	-0.46	0.643	1.00
Right ProS	-1.98	0.048	1	-1.18	0.240	1.00	-1.74	0.082	1.00
Right PeEc	1.64	0.100	1	0.33	0.741	1.00	1.17	0.241	1.00
Right STGa	0.09	0.928	1	-0.22	0.825	1.00	0.09	0.925	1.00
Right PBelt	-1.25	0.212	1	-0.64	0.521	1.00	-1.02	0.306	1.00
Right A5	-1.23	0.219	1	-1.30	0.195	1.00	-1.26	0.208	1.00
Right PHA1	-1.72	0.085	1	-1.01	0.314	1.00	-1.38	0.168	1.00
Right PHA3	-1.65	0.100	1	-1.73	0.085	1.00	-1.75	0.081	1.00
Right STSda	-0.53	0.594	1	-0.77	0.439	1.00	-0.66	0.508	1.00
Right STSdp	-1.04	0.299	1	-1.12	0.264	1.00	-1.14	0.256	1.00
Right STSvp	-0.99	0.320	1	-0.67	0.504	1.00	-0.96	0.338	1.00
Right TGd	-0.88	0.378	1	-1.49	0.137	1.00	-1.25	0.211	1.00
Right TE1a	0.16	0.871	1	-0.40	0.689	1.00	-0.06	0.955	1.00
Right TE1p	-0.82	0.415	1	-0.53	0.597	1.00	-0.74	0.458	1.00
Right TE2a	-0.33	0.740	1	-1.07	0.283	1.00	-0.64	0.525	1.00

Right TF	-0.63	0.532	1	-0.76	0.446	1.00	-0.65	0.513	1.00
Right TE2p	-0.91	0.361	1	-1.10	0.273	1.00	-1.00	0.317	1.00
Right PHT	-0.09	0.929	1	-0.04	0.966	1.00	0.01	0.994	1.00
Right PH	-0.78	0.436	1	-1.22	0.225	1.00	-0.91	0.364	1.00
Right TPOJ1	-1.91	0.056	1	-1.28	0.201	1.00	-1.66	0.097	1.00
Right TPOJ2	-2.29	0.022	1	-1.34	0.181	1.00	-2.06	0.040	1.00
Right TPOJ3	-1.56	0.119	1	-0.34	0.732	1.00	-1.05	0.292	1.00
Right DVT	-1.91	0.057	1	-1.47	0.142	1.00	-1.90	0.058	1.00
Right PGp	-1.26	0.209	1	-0.33	0.743	1.00	-0.80	0.426	1.00
Right IP2	-1.09	0.275	1	-0.81	0.416	1.00	-0.97	0.331	1.00
Right IP1	-1.19	0.234	1	-0.92	0.356	1.00	-1.16	0.246	1.00
Right IP0	-0.93	0.353	1	-0.65	0.515	1.00	-0.96	0.337	1.00
Right PFop	-1.84	0.067	1	-1.46	0.144	1.00	-1.64	0.101	1.00
Right PF	-1.89	0.060	1	-0.80	0.422	1.00	-1.47	0.141	1.00
Right PFm	-1.23	0.218	1	-1.03	0.301	1.00	-1.22	0.224	1.00
Right PGi	-1.45	0.148	1	-0.92	0.359	1.00	-1.29	0.197	1.00
Right PGs	-1.02	0.306	1	-0.70	0.487	1.00	-0.99	0.324	1.00
Right V6A	-0.58	0.563	1	-0.29	0.776	1.00	-0.39	0.697	1.00
Right VMV1	-2.33	0.020	1	-1.76	0.080	1.00	-2.27	0.024	1.00
Right VMV3	-2.92	0.004	1	-1.88	0.061	1.00	-2.68	0.008	1.00
Right PHA2	-1.32	0.188	1	-1.20	0.229	1.00	-1.19	0.234	1.00
Right V4t	-0.77	0.441	1	-0.71	0.478	1.00	-0.74	0.459	1.00
Right FST	-1.81	0.071	1	-1.08	0.282	1.00	-1.57	0.117	1.00
Right V3CD	-0.87	0.385	1	-0.62	0.532	1.00	-0.62	0.535	1.00
Right LO3	-1.98	0.049	1	-0.96	0.339	1.00	-1.53	0.127	1.00
Right VMV2	-2.77	0.006	1	-1.93	0.054	1.00	-2.68	0.007	1.00
Right 31pd	-0.87	0.382	1	-1.12	0.263	1.00	-1.12	0.261	1.00
Right 31a	-0.68	0.498	1	-0.97	0.330	1.00	-0.93	0.350	1.00
Right VVC	-2.09	0.037	1	-1.61	0.108	1.00	-2.00	0.046	1.00
Right 25	-0.28	0.783	1	-0.76	0.445	1.00	-0.55	0.583	1.00
Right s32	-0.62	0.535	1	-1.18	0.239	1.00	-0.79	0.427	1.00
Right pOFC	0.99	0.321	1	0.28	0.780	1.00	0.72	0.472	1.00
Right PoI1	0.02	0.982	1	-0.22	0.823	1.00	-0.11	0.914	1.00
Right Ig	-1.65	0.099	1	-1.75	0.080	1.00	-1.90	0.058	1.00
Right FOP5	-1.16	0.246	1	-1.84	0.067	1.00	-1.45	0.148	1.00
Right p10p	-0.49	0.626	1	-0.69	0.487	1.00	-0.46	0.646	1.00
Right p47r	-0.93	0.353	1	-1.78	0.075	1.00	-1.40	0.163	1.00
Right TGv	-0.84	0.401	1	-1.13	0.257	1.00	-0.89	0.373	1.00
Right MBelt	-2.56	0.011	1	-1.53	0.128	1.00	-2.05	0.041	1.00
Right LBelt	-1.70	0.089	1	-0.68	0.496	1.00	-1.27	0.203	1.00

Right A4	0.08	0.937	1	-0.24	0.809	1.00	0.04	0.972	1.00
Right STSva	0.39	0.700	1	-0.42	0.671	1.00	0.05	0.956	1.00
Right TE1m	-0.30	0.763	1	-0.96	0.337	1.00	-0.72	0.469	1.00
Right PI	-0.30	0.766	1	-0.45	0.653	1.00	-0.43	0.664	1.00
Right a32pr	-0.75	0.455	1	-1.27	0.203	1.00	-0.93	0.351	1.00
Right p24	0.20	0.840	1	-0.54	0.588	1.00	0.02	0.982	1.00
Left V1	-0.76	0.449	1	-0.29	0.774	1.00	-0.51	0.607	1.00
Left MST	-2.14	0.032	1	-1.50	0.133	1.00	-2.04	0.042	1.00
Left V6	-0.90	0.368	1	-0.17	0.862	1.00	-0.44	0.662	1.00
Left V2	-1.44	0.149	1	-1.05	0.296	1.00	-1.33	0.185	1.00
Left V3	-1.40	0.163	1	-1.23	0.218	1.00	-1.38	0.169	1.00
Left V4	-1.76	0.078	1	-1.87	0.062	1.00	-1.87	0.062	1.00
Left V8	-1.58	0.115	1	-1.84	0.066	1.00	-1.79	0.073	1.00
Left 4	-0.49	0.627	1	-1.26	0.209	1.00	-0.78	0.436	1.00
Left 3b	0.58	0.564	1	0.00	0.998	1.00	0.45	0.653	1.00
Left FEF	-0.18	0.854	1	-1.32	0.186	1.00	-0.59	0.557	1.00
Left PEF	0.13	0.893	1	-0.30	0.768	1.00	0.12	0.908	1.00
Left 55b	0.49	0.623	1	-0.65	0.513	1.00	0.12	0.901	1.00
Left V3A	-0.80	0.425	1	-0.15	0.882	1.00	-0.54	0.592	1.00
Left RSC	-0.68	0.495	1	-0.77	0.439	1.00	-0.60	0.552	1.00
Left POS2	-1.66	0.097	1	-1.15	0.250	1.00	-1.54	0.124	1.00
Left V7	-0.66	0.509	1	0.58	0.561	1.00	-0.04	0.964	1.00
Left IPS1	-0.55	0.580	1	-1.14	0.256	1.00	-0.95	0.342	1.00
Left FFC	-1.84	0.066	1	-2.18	0.030	1.00	-2.17	0.030	1.00
Left V3B	-1.31	0.191	1	-0.73	0.464	1.00	-1.15	0.251	1.00
Left LO1	-1.46	0.145	1	-1.61	0.107	1.00	-1.50	0.133	1.00
Left LO2	-1.60	0.109	1	-1.36	0.174	1.00	-1.48	0.140	1.00
Left PIT	-1.43	0.153	1	-1.71	0.087	1.00	-1.62	0.105	1.00
Left MT	-2.31	0.021	1	-2.11	0.035	1.00	-2.34	0.019	1.00
Left A1	-2.15	0.032	1	-1.07	0.286	1.00	-1.75	0.080	1.00
Left PSL	-0.07	0.945	1	-0.59	0.554	1.00	-0.40	0.688	1.00
Left SFL	-0.27	0.788	1	-0.82	0.412	1.00	-0.34	0.736	1.00
Left PCV	-1.05	0.295	1	-0.98	0.328	1.00	-1.13	0.261	1.00
Left STV	-1.44	0.149	1	-1.19	0.234	1.00	-1.45	0.147	1.00
Left 7Pm	-1.77	0.076	1	-1.03	0.304	1.00	-1.59	0.112	1.00
Left 7m	-1.39	0.165	1	-0.98	0.326	1.00	-1.29	0.198	1.00
Left POS1	-1.81	0.070	1	-2.00	0.046	1.00	-2.08	0.038	1.00
Left 23d	-1.64	0.101	1	-0.94	0.347	1.00	-1.40	0.161	1.00
Left v23ab	-1.49	0.136	1	-1.76	0.079	1.00	-1.72	0.086	1.00
Left d23ab	-1.05	0.293	1	-1.45	0.147	1.00	-1.24	0.216	1.00

Left 31pv	-0.61	0.545	1	-1.05	0.296	1.00	-0.91	0.365	1.00
Left 5m	-0.49	0.622	1	-1.62	0.105	1.00	-1.14	0.255	1.00
Left 5mv	-1.32	0.187	1	-1.83	0.068	1.00	-1.66	0.098	1.00
Left 23c	-0.54	0.591	1	-1.45	0.146	1.00	-1.01	0.311	1.00
Left 5L	-1.68	0.094	1	-1.51	0.132	1.00	-1.85	0.065	1.00
Left 24dd	-1.70	0.090	1	-2.64	0.008	1.00	-2.21	0.028	1.00
Left 24dv	-1.19	0.236	1	-1.86	0.063	1.00	-1.54	0.124	1.00
Left 7AL	-1.22	0.221	1	-1.31	0.191	1.00	-1.28	0.202	1.00
Left SCEF	-0.79	0.428	1	-0.97	0.334	1.00	-0.77	0.439	1.00
Left 6ma	-0.30	0.761	1	-1.06	0.287	1.00	-0.58	0.560	1.00
Left 7Am	-1.00	0.320	1	-1.12	0.264	1.00	-1.13	0.260	1.00
Left 7PL	0.06	0.949	1	-0.87	0.387	1.00	-0.55	0.585	1.00
Left 7PC	-0.87	0.382	1	-1.60	0.110	1.00	-1.46	0.144	1.00
Left LIPv	-1.39	0.165	1	-1.57	0.118	1.00	-1.58	0.114	1.00
Left VIP	-1.33	0.185	1	-1.27	0.206	1.00	-1.45	0.148	1.00
Left MIP	-1.01	0.314	1	-0.73	0.464	1.00	-0.98	0.328	1.00
Left 1	-0.15	0.882	1	-0.83	0.409	1.00	-0.40	0.689	1.00
Left 2	-0.83	0.405	1	-1.23	0.220	1.00	-0.98	0.326	1.00
Left 3a	0.72	0.473	1	-0.35	0.724	1.00	0.35	0.728	1.00
Left 6d	-0.02	0.986	1	-1.20	0.229	1.00	-0.37	0.709	1.00
Left 6mp	-1.29	0.197	1	-1.40	0.161	1.00	-1.36	0.175	1.00
Left 6v	0.14	0.891	1	-1.42	0.155	1.00	-0.58	0.564	1.00
Left p24pr	0.05	0.957	1	-0.55	0.581	1.00	-0.17	0.865	1.00
Left 33pr	-0.01	0.990	1	-0.06	0.950	1.00	0.22	0.827	1.00
Left a24pr	0.06	0.950	1	-0.71	0.477	1.00	-0.22	0.823	1.00
Left p32pr	-0.53	0.598	1	-1.53	0.127	1.00	-0.98	0.329	1.00
Left a24	0.88	0.377	1	-0.55	0.580	1.00	0.40	0.690	1.00
Left d32	-0.40	0.689	1	-0.99	0.323	1.00	-0.61	0.540	1.00
Left 8BM	-0.37	0.711	1	-0.60	0.551	1.00	-0.41	0.681	1.00
Left p32	-0.28	0.780	1	-0.90	0.370	1.00	-0.54	0.587	1.00
Left 10r	-0.37	0.708	1	-1.37	0.172	1.00	-0.91	0.361	1.00
Left 47m	-0.51	0.609	1	-1.41	0.159	1.00	-0.90	0.367	1.00
Left 8Av	0.77	0.439	1	-0.47	0.642	1.00	0.46	0.644	1.00
Left 8Ad	-0.25	0.806	1	-0.77	0.442	1.00	-0.40	0.686	1.00
Left 9m	-0.25	0.803	1	-0.48	0.630	1.00	-0.34	0.736	1.00
Left 8BL	-0.33	0.741	1	-0.50	0.620	1.00	-0.27	0.789	1.00
Left 9p	0.56	0.576	1	-0.06	0.952	1.00	0.41	0.680	1.00
Left 10d	0.18	0.859	1	-0.05	0.957	1.00	0.18	0.860	1.00
Left 8C	0.84	0.403	1	-0.31	0.756	1.00	0.55	0.583	1.00
Left 44	0.44	0.662	1	-0.90	0.369	1.00	-0.17	0.863	1.00

Left 45	-0.01	0.993	1	-1.42	0.155	1.00	-0.70	0.486	1.00
Left 47l	-0.50	0.614	1	-2.20	0.028	1.00	-1.37	0.170	1.00
Left a47r	1.18	0.240	1	-0.68	0.497	1.00	0.42	0.672	1.00
Left 6r	0.56	0.577	1	-1.18	0.239	1.00	-0.20	0.839	1.00
Left IFJa	0.12	0.904	1	-0.47	0.636	1.00	-0.03	0.975	1.00
Left IFJp	0.47	0.636	1	-0.67	0.502	1.00	0.00	0.997	1.00
Left IFSp	0.21	0.837	1	-1.27	0.205	1.00	-0.37	0.710	1.00
Left IFSa	0.21	0.837	1	-1.16	0.247	1.00	-0.42	0.672	1.00
Left p9-46v	0.95	0.341	1	-0.21	0.830	1.00	0.57	0.568	1.00
Left 46	0.20	0.839	1	-0.46	0.649	1.00	0.07	0.941	1.00
Left a9-46v	1.20	0.229	1	-0.11	0.913	1.00	0.83	0.407	1.00
Left 9-46d	-0.08	0.934	1	-0.37	0.713	1.00	-0.14	0.885	1.00
Left 9a	0.36	0.721	1	0.02	0.984	1.00	0.32	0.746	1.00
Left 10v	-0.57	0.570	1	-1.81	0.071	1.00	-1.25	0.212	1.00
Left a10p	0.72	0.474	1	-0.61	0.541	1.00	0.17	0.863	1.00
Left 10pp	-0.23	0.815	1	-1.75	0.080	1.00	-1.07	0.283	1.00
Left 11l	0.40	0.687	1	-1.45	0.149	1.00	-0.50	0.616	1.00
Left 13l	0.01	0.996	1	-1.15	0.249	1.00	-0.62	0.533	1.00
Left OFC	0.17	0.867	1	-1.10	0.271	1.00	-0.44	0.661	1.00
Left 47s	-0.59	0.555	1	-1.29	0.197	1.00	-0.99	0.323	1.00
Left LIPd	-0.78	0.438	1	-1.28	0.200	1.00	-0.99	0.322	1.00
Left 6a	-0.06	0.956	1	-0.85	0.395	1.00	-0.29	0.773	1.00
Left i6-8	0.76	0.448	1	-0.69	0.493	1.00	0.38	0.707	1.00
Left s6-8	0.53	0.596	1	-0.38	0.707	1.00	0.33	0.745	1.00
Left 43	-0.34	0.731	1	-2.17	0.030	1.00	-1.34	0.180	1.00
Left OP4	-0.82	0.415	1	-1.77	0.076	1.00	-1.42	0.157	1.00
Left OP1	-1.09	0.277	1	-2.17	0.030	1.00	-1.72	0.087	1.00
Left OP2-3	-0.24	0.809	1	-1.31	0.192	1.00	-0.71	0.479	1.00
Left 52	-1.91	0.057	1	-2.22	0.027	1.00	-2.22	0.027	1.00
Left RI	-1.39	0.166	1	-1.90	0.058	1.00	-1.72	0.086	1.00
Left PFcm	-1.42	0.155	1	-1.54	0.124	1.00	-1.62	0.106	1.00
Left PoI2	0.12	0.905	1	-0.77	0.440	1.00	-0.34	0.732	1.00
Left TA2	-0.60	0.551	1	-1.25	0.210	1.00	-0.89	0.372	1.00
Left FOP4	-0.12	0.901	1	-1.27	0.205	1.00	-0.69	0.492	1.00
Left MI	-0.21	0.837	1	-1.20	0.230	1.00	-0.64	0.523	1.00
Left Pir	-0.17	0.862	1	-0.28	0.777	1.00	-0.26	0.795	1.00
Left AVI	-0.22	0.825	1	-1.25	0.212	1.00	-0.71	0.480	1.00
Left AAIC	-0.22	0.824	1	-0.77	0.441	1.00	-0.54	0.590	1.00
Left FOP1	0.58	0.560	1	-1.18	0.238	1.00	-0.27	0.790	1.00
Left FOP3	-0.17	0.868	1	-1.07	0.286	1.00	-0.59	0.553	1.00

Left FOP2	0.10	0.919	1	-1.12	0.262	1.00	-0.48	0.631	1.00
Left PFt	-0.34	0.730	1	-0.85	0.398	1.00	-0.54	0.588	1.00
Left AIP	-0.33	0.740	1	-0.80	0.424	1.00	-0.56	0.577	1.00
Left EC	-0.31	0.756	1	-1.25	0.211	1.00	-0.59	0.556	1.00
Left PreS	0.30	0.761	1	0.36	0.718	1.00	0.55	0.586	1.00
Left H	0.32	0.752	1	-0.77	0.444	1.00	-0.13	0.895	1.00
Left ProS	-1.12	0.263	1	-0.93	0.351	1.00	-1.05	0.295	1.00
Left PeEc	-0.44	0.657	1	-1.31	0.192	1.00	-0.83	0.409	1.00
Left STGa	-0.39	0.699	1	-1.75	0.080	1.00	-1.16	0.247	1.00
Left PBelt	-0.77	0.439	1	-1.45	0.147	1.00	-1.05	0.294	1.00
Left A5	-0.09	0.926	1	-1.47	0.141	1.00	-0.75	0.451	1.00
Left PHA1	-0.25	0.800	1	-0.09	0.928	1.00	0.15	0.883	1.00
Left PHA3	-0.98	0.329	1	-1.53	0.127	1.00	-1.24	0.216	1.00
Left STSda	-0.65	0.518	1	-2.34	0.020	1.00	-1.67	0.096	1.00
Left STSdp	-0.50	0.617	1	-1.87	0.061	1.00	-1.21	0.225	1.00
Left STSvp	-0.75	0.455	1	-2.06	0.040	1.00	-1.54	0.124	1.00
Left TGd	-0.40	0.689	1	-2.62	0.009	1.00	-1.50	0.134	1.00
Left TE1a	-0.17	0.867	1	-2.58	0.010	1.00	-1.54	0.124	1.00
Left TE1p	-0.55	0.581	1	-0.99	0.321	1.00	-0.80	0.427	1.00
Left TE2a	0.52	0.602	1	-0.77	0.443	1.00	0.01	0.995	1.00
Left TF	0.11	0.911	1	-1.18	0.240	1.00	-0.49	0.624	1.00
Left TE2p	-1.75	0.081	1	-1.30	0.193	1.00	-1.60	0.111	1.00
Left PHT	-1.79	0.074	1	-1.60	0.111	1.00	-1.85	0.064	1.00
Left PH	-1.60	0.110	1	-1.68	0.093	1.00	-1.73	0.085	1.00
Left TPOJ1	-0.88	0.379	1	-1.94	0.053	1.00	-1.43	0.154	1.00
Left TPOJ2	-2.33	0.020	1	-1.98	0.048	1.00	-2.39	0.017	1.00
Left TPOJ3	-1.92	0.056	1	-1.50	0.134	1.00	-1.76	0.078	1.00
Left DVT	-1.62	0.105	1	-1.46	0.144	1.00	-1.71	0.087	1.00
Left PGp	-0.81	0.419	1	-0.81	0.419	1.00	-0.84	0.404	1.00
Left IP2	-0.63	0.526	1	-0.76	0.448	1.00	-0.71	0.475	1.00
Left IP1	-0.42	0.673	1	-0.18	0.857	1.00	-0.37	0.714	1.00
Left IP0	-1.02	0.309	1	-0.21	0.833	1.00	-0.69	0.488	1.00
Left PFop	-0.19	0.845	1	-1.49	0.136	1.00	-0.79	0.429	1.00
Left PF	-0.35	0.727	1	-1.00	0.317	1.00	-0.66	0.512	1.00
Left PFm	-0.37	0.708	1	-0.35	0.728	1.00	-0.36	0.719	1.00
Left PGi	-1.20	0.232	1	-1.18	0.239	1.00	-1.24	0.217	1.00
Left PGs	-0.79	0.432	1	-0.64	0.519	1.00	-0.82	0.414	1.00
Left V6A	-0.27	0.789	1	-0.40	0.688	1.00	-0.38	0.703	1.00
Left VMV1	-1.24	0.215	1	-1.58	0.114	1.00	-1.42	0.156	1.00
Left VMV3	-1.63	0.103	1	-1.75	0.081	1.00	-1.76	0.078	1.00

Left PHA2	-0.67	0.505	1	-0.82	0.411	1.00	-0.58	0.560	1.00
Left V4t	-1.74	0.082	1	-1.31	0.190	1.00	-1.54	0.125	1.00
Left FST	-1.43	0.152	1	-1.03	0.305	1.00	-1.31	0.189	1.00
Left V3CD	-1.78	0.076	1	-1.50	0.135	1.00	-1.57	0.118	1.00
Left LO3	-2.16	0.031	1	-1.88	0.060	1.00	-2.11	0.035	1.00
Left VMV2	-1.47	0.141	1	-2.02	0.043	1.00	-1.87	0.062	1.00
Left 31pd	-0.98	0.329	1	-1.19	0.234	1.00	-1.16	0.248	1.00
Left 31a	-1.23	0.218	1	-1.73	0.084	1.00	-1.59	0.112	1.00
Left VVC	-2.24	0.025	1	-2.66	0.008	1.00	-2.62	0.009	1.00
Left 25	0.03	0.973	1	0.49	0.626	1.00	0.41	0.683	1.00
Left s32	0.44	0.663	1	-0.47	0.642	1.00	0.00	0.996	1.00
Left pOFC	0.56	0.577	1	-0.04	0.972	1.00	0.32	0.746	1.00
Left PoI1	0.05	0.962	1	-1.45	0.148	1.00	-0.72	0.469	1.00
Left Ig	-0.99	0.320	1	-1.43	0.152	1.00	-1.21	0.227	1.00
Left FOP5	-0.64	0.526	1	-1.77	0.078	1.00	-1.12	0.264	1.00
Left p10p	0.01	0.988	1	-0.31	0.757	1.00	-0.05	0.960	1.00
Left p47r	0.54	0.589	1	-0.79	0.432	1.00	0.00	0.998	1.00
Left TGv	-0.75	0.454	1	-2.55	0.011	1.00	-1.66	0.097	1.00
Left MBelt	-2.84	0.005	1	-2.26	0.024	1.00	-2.82	0.005	1.00
Left LBelt	-1.40	0.162	1	-1.01	0.312	1.00	-1.19	0.235	1.00
Left A4	-0.32	0.747	1	-1.82	0.069	1.00	-1.09	0.276	1.00
Left STSva	-0.30	0.764	1	-2.47	0.014	1.00	-1.52	0.128	1.00
Left TE1m	0.08	0.936	1	-1.68	0.094	1.00	-0.79	0.431	1.00
Left PI	0.31	0.757	1	-1.03	0.304	1.00	-0.29	0.775	1.00
Left a32pr	-0.47	0.638	1	-1.34	0.182	1.00	-0.90	0.366	1.00
Left p24	-0.38	0.706	1	-0.73	0.467	1.00	-0.47	0.642	1.00
